# Special Issue “Viral Infections, Host Immunity and Vaccines”

**DOI:** 10.3390/vaccines14040332

**Published:** 2026-04-09

**Authors:** Sarah N. Mueni, Henry M. Kariithi, Salman L. Butt

**Affiliations:** 1 Department of Biochemistry, Jomo Kenyatta University of Agriculture and Technology, Juja Kwale Road, Nairobi P.O. Box 62000-00200, Kenya; sarah.mueni@jkuat.ac.ke; 2 Biotechnology Research Institute, Kenya Agricultural and Livestock Research Organization, Kikuyu P.O. Box 362-00902, Kenya; henry.kariithi@kalro.org; 3 Department of Population Medicine and Diagnostic Sciences, College of Veterinary Medicine, Cornell University, Ithaca, NY 14853, USA

## 1. Introduction

Viral infections remain among the most persistent and evolving threats to global health [1]. Their outcome and the ultimate success of vaccines hinge on a complex interplay between viral evolution, the host immune response, and the environmental and population contexts of the encounter [2]. Viruses have become remarkably adept at evading immunity, while subtle genetic shifts continue to reshape transmission dynamics and challenge vaccine effectiveness [3]. In this context, a comprehensive understanding of host immunity, from molecular mechanisms of immune evasion to population-level immunity and immunological memory, is essential to improving disease control strategies [4]. Yet important gaps remain, particularly in linking mechanistic models to real-world vaccine performance and understanding how these processes unfold across different hosts and settings [5]. Insights from experimental models and cross-species studies are equally important in terms of refining our understanding of viral pathogenesis and vaccine performance [6].

The six contributions in this Special Issue offer a multidisciplinary window onto these questions, spanning experimental studies, genomic analyses, and conceptual reviews. These contributions cover diverse aspects of viral immunity and vaccination, including immune-evasion strategies of important veterinary viruses that inform improved live attenuated vaccines design [7], and shifting dengue genotypes in the Americas with implications for vaccine effectiveness [8]. They also cover serosurveys identifying gaps in measles, mumps, and rubella immunity in Kyrgyzstan [9], alongside analyses of the dual-edged role of memory cells in protection and autoimmunity [10]. Additional studies examine whether bovine viral diarrhea virus (BVDV) vaccines elicit measurable immune responses in white-tailed deer [11], and how differences in inoculation volume influence influenza outcomes in mice [12]. Each paper adds a distinct but complementary piece to the puzzle, collectively helping to bridge the gap between molecular mechanisms, population-level observations, and practical vaccine considerations. Taken together, they reinforce the principle that effective viral control demands integrated perspectives across disciplines and systems. We hope this collection will stimulate further research, highlight remaining challenges, and contribute to the development of more effective vaccines and surveillance strategies.

## 2. Overview of Contributions to the Special Issue

### 2.1. Vaccine-Induced Humoral and Cellular Immunity in a Wildlife Host

Recognizing that infection outcomes and vaccine success ultimately depend on how the host immune system responds, Boggiatto and colleagues [11] explored this question in a species that rarely receives commercial livestock vaccines, white-tailed deer. Their study aimed to assess whether BVDV vaccines used in cattle could reliably stimulate measurable immune responses in deer, and compared two widely available formulations: killed-virus (KV) and modified live-virus (MLV) vaccines. They tracked both arms of the adaptive response—virus-neutralizing antibodies and BVDV-specific T-cell activity measured through interferon gamma (IFN-γ) production. On the humoral side, the deer developed clear neutralizing antibody responses after vaccination and boosting. The MLV vaccine worked faster and more potently, generating antibodies against both BVDV serotypes 1 and 2. In contrast, the KV vaccine mainly elicited antibodies against BVDV-1, and only after the booster. When it came to cellular immunity, however, the picture was more nuanced. Using a highly sensitive PrimeFlow RNA assay combined with flow cytometry, the team looked for antigen-specific IFN-γ mRNA expression in T-cell subsets. Despite this sophisticated approach—and despite IFN-γ’s well-known role as a central coordinator of antiviral cellular responses—no detectable BVDV-specific T-cell responses appeared after stimulation.

Taken together, the work shows that commercial cattle BVDV vaccines can reliably trigger measurable antibody responses in white-tailed deer, with the modified live version performing noticeably better. At the same time, it underscores how challenging it can be to capture cell-mediated immunity in non-traditional species where species-specific reagents are scarce. The successful application of the PrimeFlow RNA assay in deer represents a genuine methodological step forward. The findings carry broader significance. They suggest that livestock vaccines could have real value in wildlife management, particularly for species that sit at the interface of agricultural and natural ecosystems and may play roles in zoonotic transmission cycles. Future studies will need to fine-tune sampling times and stimulation conditions to better understand the cellular response. But this paper already gives us a clearer roadmap toward protecting deer, and perhaps other wildlife—with tools already sitting on the shelf.

### 2.2. Viral Immune Evasion as a Blueprint for Live Attenuated Vaccine Design

Animal viruses have evolved a remarkable ability to evade host immune defenses, a theme brought together by Chen and Zhang [7] in a wide-ranging review that maps the molecular strategies used to sabotage innate antiviral immunity and guide the design of next-generation live attenuated vaccines. At its core, the innate immune system is the body’s rapid-response force, pattern-recognition receptors act like sentinels, spotting viral components and kicking off signaling cascades that trigger interferon production and a broad antiviral state. Yet many viruses have developed sophisticated ways to disrupt, circumvent, or suppress these defenses, giving themselves time to replicate and establish infection.

The review zeroes in on several major veterinary pathogens that cause serious economic losses. Take African swine fever virus, which packs multiple immune antagonists that interfere with DNA sensing. One protein, QP383R, directly blocks the DNA sensor cGAS, while others—including B175L and members of the MGF360 and MGF505 families—shut down STING signaling and dampen interferon output. Foot-and-mouth disease virus (FMDV) takes a different route, using its proteases Lpro and 3Cpro to dismantle antiviral signaling and prevent key transcription factors from switching on interferon genes. RNA viruses play similar games in the cytoplasm. Porcine reproductive and respiratory syndrome virus deploys non-structural proteins such as Nsp3 and Nsp11 to interfere with RIG-I and MDA5, silencing interferon induction. Pseudorabies virus goes after transcription factors like IRF3 with proteins including UL13, UL41, and US3, breaking the signaling chain. Some viruses go even further, targeting restriction factors and interferon pathways themselves. FMDV’s Lpro, for example, suppresses NF-κB and cleaves STAT1 and STAT2, crippling the interferon response. Avian influenza virus uses NS1, PB1, and PB2 to hit MAVS, IKK kinases, and JAK1, weakening the entire antiviral network.

Beyond direct signaling sabotage, certain viruses reshape the cellular environment to their advantage—blocking stress granule formation, tweaking apoptosis, or rewiring host metabolism and regulatory RNA networks. These layered strategies reveal just how deeply viruses have learned to manipulate their hosts. This review emphasizes that many of these immune-evasion proteins double as virulence factors, and that deleting or modifying them can markedly attenuate viral pathogenicity while preserving its ability to stimulate strong immunity. This insight offers a practical roadmap toward the design of safer, more effective live attenuated vaccines against some of the most damaging animal viruses we face.

### 2.3. Memory Immune Cells at the Interface of Protection and Autoimmunity

Immunological memory, one of the hallmarks of adaptive immunity, enables faster responses upon re-exposure to pathogens and underpins vaccination [13]. In their comprehensive review, Giri and Batra [10] examine the cellular and molecular basis of memory immune cells and their dual role in protection and autoimmunity. Memory T and B cells are the long-term sentinels of the immune system. Upon re-encountering a familiar pathogen, they unleash rapid, high-magnitude responses. Yet the same persistence and heightened reactivity can turn against self-tissues, turning protectors into contributors to chronic immunopathology. The authors carefully dissect the major memory T-cell subsets. Central memory T cells (TCM) reside mainly in lymphoid organs and possess strong proliferative potential; they expand vigorously on antigen re-exposure and are particularly important in latent tuberculosis, where higher frequencies of IFN-γ- and TNF-α-producing TCMs distinguish controlled infection from active disease. Effector memory T cells, by contrast, patrol peripheral tissues and deliver immediate effector functions—producing interferon-γ to limit influenza replication or deploying perforin and granzymes to destroy parasite-infected hepatocytes in malaria.

Tissue-resident memory T cells (TRMs) take up permanent residence in barrier sites such as the skin and lungs, providing instant local defense against influenza and herpes simplex virus through rapid cytokine release. The darker side of this persistence is equally clear. IL-17-producing TRMs that linger in previously inflamed skin can trigger psoriasis flares years later, while synovial TRMs sustain recurrent inflammation in rheumatoid arthritis. Autoreactive memory CD4^+^ T cells that target myelin antigens in the central nervous system—expressing migration markers such as CXCR3 and ICOS—drive progression in multiple sclerosis. Similarly, memory T cells recognizing pancreatic islet antigens relentlessly destroy insulin-producing β-cells in type 1 diabetes.

Memory B cells complete the picture. They stand ready to differentiate into antibody-secreting plasma cells or to undergo further affinity maturation, broadening neutralization against evolving threats such as SARS-CoV-2 variants. In the pursuit of an effective HIV vaccine, memory B cells are emerging as central to several promising strategies, including germline-targeting approaches that guide B-cell maturation toward broadly neutralizing antibodies, as well as advances in mRNA-based platforms and CD8^+^ T cell-focused vaccine designs. Yet, when these cells become autoreactive, they fuel sustained autoantibody production in Graves’ disease (targeting the thyroid), myasthenia gravis (attacking acetylcholine receptors and causing muscle weakness), Hashimoto’s thyroiditis, autoimmune hemolytic anemia, and systemic sclerosis. Recognizing this double-edged role, the review turns to therapy. B-cell depletion with rituximab (anti-CD20), BAFF inhibitors such as belimumab, and agents that block co-stimulation or inflammatory cytokines (IL-17, IL-23) are already showing promise in dampening pathogenic memory responses in psoriasis, rheumatoid arthritis, and multiple sclerosis. Ultimately, Giri and Batra remind us that memory cells sit at the very heart of both vaccine success and autoimmune misfortune. The more precisely we understand the signals that govern their formation, maintenance, and regulation, the closer we come to designing vaccines that elicit stronger, longer-lasting protection and to immunotherapies that silence harmful memory while limiting immunopathology.

### 2.4. Evolutionary Dynamics of DENV-2 and Implications for Vaccine Matching

Dengue virus (DENV) remains a major challenge for vaccine design, as its four serotypes are genetically and antigenically distinct, and infection with one serotype does not confer full immunity against the others [14,15]. In this context, understanding the evolutionary dynamics of circulating strains is essential to assessing vaccine effectiveness and guiding disease control strategies. Lagrave et al. [8] address this by conducting a retrospective analysis of the genetic evolution of DENV-2 in the French Territories of the Americas between 2000 and 2024. Using 77 whole-genome sequences generated through Oxford Nanopore sequencing together with 122 reference sequences from GenBank, the authors reconstructed phylogenetic relationships and evolutionary trends. Their analysis showed that, until the 2019 epidemic, the Asian–American genotype had been the dominant player. After that, the Cosmopolitan genotype steadily took over, fueling the outbreaks seen between 2019 and 2024. The viruses behind the most recent 2023–2024 waves sit together in a tight, well-supported cluster and closely resemble strains previously reported in Asia—bolstering the likelihood of repeated introductions, potentially through international travel and trade.

Additionally, the authors directly compared the circulating viruses to the two licensed vaccines, Dengvaxia and Qdenga, focusing on the pre-membrane and envelope proteins—the main targets of protective antibodies. They found 42 amino acid changes relative to the Dengvaxia backbone and 46 relative to Qdenga, some of them in positions that have been associated with antigenic properties or antibody-binding.

Because the envelope protein sits at the heart of both viral entry and immune recognition, these shifts could reshape epitopes and may have implications for vaccine-induced protection. The presence of these conserved substitutions across genotypes underscores the ongoing evolutionary arms race between the virus and host immunity.

Considered as a whole, this work is a compelling reminder of how powerful genomic epidemiology has become. It does not merely map transmission routes and evolutionary history, it is a practical tool that helps assess whether today’s vaccines remain a good match for tomorrow’s viruses. The next critical steps will be sustained genomic surveillance combined with functional neutralization assays against emerging variants to help ensure control strategies keep pace with dengue’s restless evolution.

### 2.5. Population Immunity Gaps and the Challenge of Sustaining Herd Protection

Vaccination programs against highly contagious viruses have greatly reduced global disease burden, yet sustaining strong herd immunity remains a delicate and ongoing public health challenge—one that Popova and colleagues [9] examine through a large seroepidemiological study of measles, mumps, and rubella immunity in the Kyrgyz Republic. Drawing on samples from 6617 individuals across a wide age spectrum, they measured IgG antibody levels as a direct marker of population protection.

Their findings revealed important differences in immunity levels across the three vaccine-preventable diseases, which is both encouraging and cautionary. Rubella immunity stands at approximately 94.2%—comfortably close to the threshold needed to interrupt transmission (~92–95% population immunity). In contrast, measles and mumps show more modest protection, with seropositivity rates of 78.9% and 76.4%, respectively. These levels fall below thresholds required to prevent sustained viral transmission, leaving pockets of vulnerability that could enable these viruses to re-emerge in the future, highlighting potential risks of outbreaks. The data also reveal clear differences across age groups, pointing to the possible gaps in earlier vaccination coverage or the gradual waning of immunity over time. Particularly concerning is lower protection in younger populations, with reduced measles immunity in children (1–17 years) and mumps deficits in young adults (12–29 years), while children remain the most critical group. Beyond the serological numbers, the authors highlight how social, cultural, and socioeconomic realities shape the real-world success of immunization programs. Their findings serve as a clear reminder that herd immunity is never a static achievement. Hence, continuous surveillance and targeted efforts to close coverage gaps will be essential if we are to keep measles, mumps, and rubella firmly under control and prevent their resurgence in the Kyrgyz Republic and beyond.

### 2.6. Impact of Inoculation Parameters on Influenza Pathogenesis Models

Even small methodological variations can influence experimental models of viral infection, a point explored by Sun and colleagues [12] in their investigation of how differences in intranasal inoculation volume affect pathogenicity outcomes despite identical viral doses.

Mouse models remain the workhorse for studying influenza pathogenesis, immune responses, and vaccine performance, yet laboratory protocols often differ—especially the volume of liquid suspension used to deliver the virus. That seemingly trivial choice, the authors reasoned, could change how the virus spreads through the respiratory tract and ultimately skew disease outcomes and reproducibility.

To test this idea, they infected mice with a well-characterized lung-adapted strain of influenza A/Puerto Rico/8/34 (H1N1) using identical viral doses (expressed as plaque-forming units, PFUs) but three different inoculation volumes: 10 µL, 20 µL, and 40 µL. They ran the experiment at two viral loads—200 PFU (low dose) and 2000 PFU (high dose)—and followed several clear markers of disease progression: body weight loss, mortality, lung viral titers, and the severity of histopathological changes in lung tissue.

The results were striking. In the low-dose group (200 PFU), mice that received the 40 µL volume developed noticeably more severe disease—greater weight loss and more extensive lung pathology—than those given 10 µL or 20 µL. Under the higher viral burden (2000 PFU), both the 20 µL and 40 µL groups diverged significantly from the 10 µL group, suggesting that larger volumes enable the virus to reach deeper or more widespread areas of the respiratory tract.

Their findings underscore the importance of standardizing inoculation volume as a critical variable that can dramatically affect viral replication, disease severity, and the reliability of experimental outcomes. These insights are particularly relevant for studies investigating influenza pathogenesis, antiviral therapies, and vaccine evaluations, where reproducibility and accurate interpretation of host–virus interactions are essential.

## 3. Conclusions

Recent advances in viral infections and immunity have sharpened our understanding of host–pathogen interactions, vaccine-induced protection, and viral evolution. At the same time, it is becoming increasingly clear that these processes cannot be fully understood in isolation. As viral threats continue to evolve across human and animal populations, no single analysis, whether molecular, experimental, or epidemiological, is sufficient on its own. Progress, therefore, will depend on how effectively these perspectives are integrated.

Yet, important gaps persist. We still lack a complete understanding of what drives durable, balanced immune memory without tipping into immunopathology, while ongoing viral diversity and antigenic variation continue to challenge vaccine effectiveness. Uneven population immunity further underscores the limits of current coverage and long-lasting protection, and immune responses in wildlife and other non-traditional hosts remain poorly characterized. In addition, methodological inconsistencies in experimental systems continue to complicate interpretation, reproducibility, and cross-study comparability.

A recurring challenge is translation in moving from mechanistic insights to outcomes that hold across species, environments, and real-world settings. Bridging this gap will require stronger alignment between experimental design, field observations, and population-level data, as well as the identification of robust and context-relevant correlates of protection.

Looking ahead, future research will need to place greater emphasis on integration across disciplines, host systems, and scales of investigation. Continued genomic surveillance, functional studies linking viral variation to clinical outcomes, and refined experimental models will be essential. Expanding research into diverse host species—including wildlife—and ensuring careful evaluation of vaccine performance across contexts will further strengthen our ability to anticipate and respond to emerging threats.

Ultimately, advancing our knowledge of immune memory, viral evolution, and host diversity is the key to developing more durable and broadly protective vaccines. By bringing together complementary approaches and perspectives, the contributions in this Special Issue help move the field toward more holistic and responsive strategies for understanding and controlling viral infections in an ever-changing environment.
